# Structure, identification, and characterization of the RibD-enolase complex in *Francisella*

**DOI:** 10.1093/procel/pwaf045

**Published:** 2025-06-05

**Authors:** Xiaoyu Liu, Daniel L Clemens, Bai-Yu Lee, Roman Aguirre, Marcus A Horwitz, Z Hong Zhou

**Affiliations:** Department of Microbiology, Immunology and Molecular Genetics, University of California, Los Angeles (UCLA), 615 Charles E Young Drive South, Los Angeles, CA 90095, United States; The California NanoSystems Institute (CNSI), UCLA, 570 Westwood Plaza, Los Angeles, CA 90095, United States; Department of Medicine, UCLA, 10833 Le Conte Ave., Los Angeles, CA 90095, United States; Department of Medicine, UCLA, 10833 Le Conte Ave., Los Angeles, CA 90095, United States; Department of Microbiology, Immunology and Molecular Genetics, University of California, Los Angeles (UCLA), 615 Charles E Young Drive South, Los Angeles, CA 90095, United States; The California NanoSystems Institute (CNSI), UCLA, 570 Westwood Plaza, Los Angeles, CA 90095, United States; Department of Chemistry and Biochemistry, University of California, Los Angeles (UCLA), 607 Charles E. Young Drive East, Los Angeles, CA 90095, United States; Department of Microbiology, Immunology and Molecular Genetics, University of California, Los Angeles (UCLA), 615 Charles E Young Drive South, Los Angeles, CA 90095, United States; Department of Medicine, UCLA, 10833 Le Conte Ave., Los Angeles, CA 90095, United States; Department of Microbiology, Immunology and Molecular Genetics, University of California, Los Angeles (UCLA), 615 Charles E Young Drive South, Los Angeles, CA 90095, United States; The California NanoSystems Institute (CNSI), UCLA, 570 Westwood Plaza, Los Angeles, CA 90095, United States; Department of Chemistry and Biochemistry, University of California, Los Angeles (UCLA), 607 Charles E. Young Drive East, Los Angeles, CA 90095, United States

**Dear Editor,**

*Francisella tularensis* is a highly infectious bacterium that causes tularemia, a potentially fatal zoonotic disease. As few as 10 organisms via injection, or 25 via inhalation, can cause human infection, making *F. tularensis* extremely virulent (Clemens et al., 2018). Due to its high infectivity and bioterrorism concerns, the US Centers for Disease Control and Prevention (CDC) classifies it as a Tier 1 biological agent. The increasing threat of antibiotic resistance underscores the need for new prevention and treatment strategies for *Francisella* infections (Biot et al., 2020), such as protein-targeted therapies to disrupt essential bacterial processes. Here, we report the discovery, structure, and functions of an unexpected *Francisella* complex containing two proteins that play pivotal roles in metabolic pathways and offer insights into their potential as targets for the treatment or prevention of tularemia.

The first protein is called riboflavin biosynthesis protein D (RibD), whose homolog was first identified and characterized in *Escherichia coli* during studies on riboflavin (more commonly known as vitamin B_2_) biosynthesis (Richter et al., 1997). While microorganisms and plants synthesize riboflavin *de novo*, animals, including humans, lack this pathway, making riboflavin biosynthetic proteins potential antibacterial targets without toxicity to humans (Islam and Kumar, 2023). RibD is a key enzyme in this pathway, responsible for two sequential reactions: the deamination of 2,5-diamino-6-ribosyl-amino-4(3H) pyrimidinedione 5′-phosphate and the subsequent reduction of 5-amino-6-ribosylamino-2,4(1H,3H)-pyrimidinedione 5′-phosphate (Islam and Kumar, 2023). The second protein in the newly discovered two-protein *Francisella* complex is enolase, a key glycolytic enzyme first identified in yeast in the 1930s (Otto, 2016). Enolase catalyzes the conversion of 2-phospho-d-glycerate to phosphoenolpyruvate (PEP) in glycolysis and the reverse reaction in gluconeogenesis (Fukano and Kimura, 2014). In addition to its metabolic activity, enolase plays important roles in various nonmetabolic functions (Vadlamani et al., 2023). Studies on enolase inhibitors suggest potential for antibacterial therapy (Zhang et al., 2022).

While RibD and enolase have been previously reported as individual proteins functioning in separate metabolic pathways in other species (Kuhnel and Luisi, 2001; Stenmark et al., 2007), their interaction as a complex has never been documented before. In this study, we have determined the atomic structure of, and identified the chemical compositions and interactions within, a native RibD-enolase protein complex from *Francisella novicida*. We made this serendipitous discovery during a structure-based investigation of a *Francisella* virulence factor, its type VI secretion system (T6SS). In brief, protein samples purified from *F. novicida* lysate were analyzed by cryogenic electron microscopy (cryoEM). Approximately 5% of the particles from the 2D classification, which displayed C2 symmetry distinct from the dominant population (the overexpressed target protein—T6SS effector), were selected and refined, yielding a 3.05 Å cryoEM map (Fig. S1). To identify the proteins within the cryoEM map, we first ran DeepTracer (Pfab et al., 2021). Using the DeepTracer output, a BLAST analysis against the *F. novicida* protein database in Uniprot identified the proteins as RibD and enolase, with a molecular weight of 39.6 kDa and 49.5 kDa, respectively.

The cryoEM structure reveals a heterotetrametric complex consisting of a RibD dimer and an enolase dimer, in a dimer-of-dimer configuration (Fig. 1A and 1B). The RibD dimer adopts a roof-like conformation, sheltering, and interacting with the enolase dimer to form a closed ring, with overall dimensions of 105 Å × 110 Å × 50 Å. RibD is a bifunctional enzyme with deaminase and reductase activities, comprising an N-terminal deaminase domain (1–146) and a C-terminal reductase domain (150–350), connected by a linker region (Fig. 1C). The deaminase domain contains a β-sheet core formed by five mixed β-strands, surrounded by α-helices. The reductase domain is primarily composed of a β-sheet with seven parallel strands and a C-terminal β-hairpin, accompanied by several surrounding α-helices. Enolase consists of an N-terminal domain (1–135) with an antiparallel β-sheet of three strands and four α-helices and a C-terminal domain (141–429) with a mixed α/β-barrel (Fig. 1C). The deaminase domain of RibD interacts with enolase, forming a key interface within the complex (Fig. 1C). The reductase domain is positioned at the distal end of the RibD-enolase heterodimeric interface. This structure represents the first and direct evidence of RibD and enolase forming a complex in *Francisella*.

**Figure 1.**
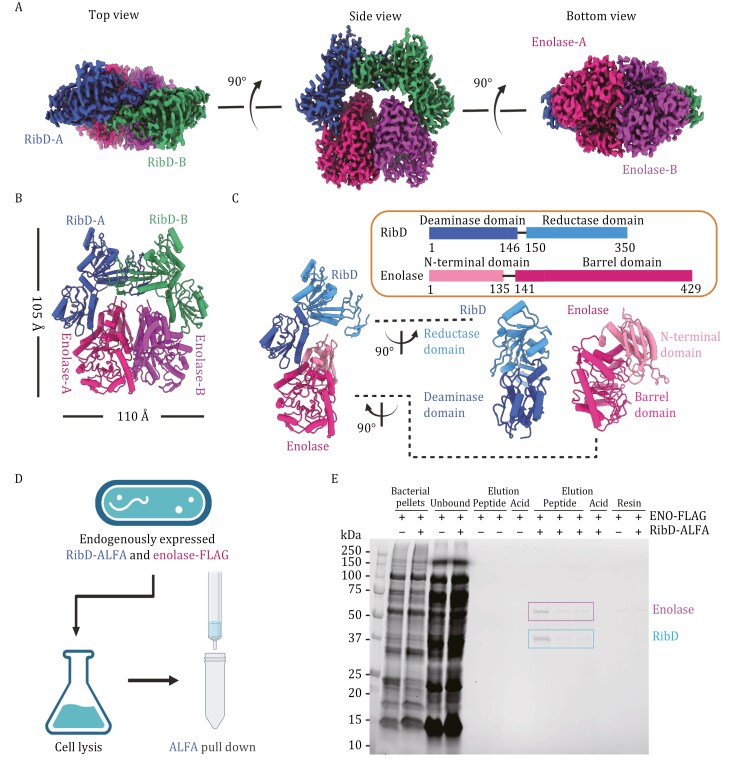
**RibD and enolase form a complex in *Francisella*.** (A) CryoEM structure of RibD-enolase complex. Three different views of the cryoEM density map of RibD-enolase. (B) Structure of RibD-enolase complex depicted in cylinder representation. (C) Domain organization of RibD and enolase. (D) Experimental flowchart for pull down assay. (E) RibD-ALFA and enolase-FLAG are pulled down by anti-ALFA resin and both are eluted by ALFA peptide. Two sets of elution of anti-ALFA resin are shown: one set corresponding to the *F. novicida* strain expressing both ENO-FLAG and RibD-ALFA and the other set corresponding to the strain expressing ENO-FLAG but not RibD-ALFA, respectively. Each set shows 2 lanes (ENO-FLAG with untagged RibD) or 3 lanes (ENO-FLAG + RibD-ALFA) corresponding to consecutive elutions of resin with the ALFA-peptide followed by a lane showing the elution with glycine HCl, pH 2.2.

To verify whether RibD and enolase interact *in vivo*, we performed pull-down assays on *F. novicida* cell lysates (Fig. 1D). We prepared *F. novicida* strains expressing FLAG-tagged enolase and ALFA-tagged RibD, along with a control strain expressing FLAG-tagged enolase without any epitope tag on the RibD. All the proteins were endogenously expressed from their native loci. Anti-ALFA pull-down was performed for lysates of both strains. SDS-PAGE (sodium dodecyl sulfate-polyacrylamide gel electrophoresis) analysis revealed two bands corresponding to the molecular weights of enolase and RibD in the strain expressing both proteins with epitope tags. In contrast, no pulldown of enolase was observed in the strain that does not express ALFA-tagged RibD (Fig. 1E). We further confirmed the two bands as enolase and RibD, respectively, by Western blotting utilizing anti-FLAG and anti-ALFA antibodies (Fig. S2A and S2B). These results demonstrate that RibD and enolase form a complex *in vivo*. In addition, anti-ALFA Western blot showed that the anti-ALFA resin pulls down 100% of the ALFA-tagged RibD but only a small percentage of the total enolase-FLAG (i.e., FLAG-enolase signals are extremely strong in the “unbound lanes” of Fig. S2A, whereas there is no signal corresponding to RibD-ALFA in the “unbound lanes” of Fig. S2B). Thus, in *F. novicida*, RibD exists only in complex with enolase, whereas the majority of enolase is not in complex with RibD. Densitometry of the enolase and RibD bands confirmed that the two proteins are present in a 1:1 ratio, consistent with that revealed in the cryoEM structure. We further expressed *F. novicida* RibD and enolase as fusions with adenylate cyclase T18 and T25 domains in *E. coli*, and we confirmed the interactions of RibD and enolase by bacterial two-hybrid assay. This assay also demonstrated enolase-enolase and RibD-RibD self-interactions (Fig. S2C and S2D).

Next, we analyzed subunit interactions (Fig. 2A–D) to understand the structural basis of assembly, starting with a focus on the RibD-enolase interface. The N-terminus of RibD helix α1 inserts into a hydrophobic groove on enolase, primarily formed by residues F350, A354, M136, M353, T384, and C363 (Fig. 2C). RibD residues M1, N3, I4, Y7, and Y8 establish extensive hydrophobic contacts, while electrostatic interactions between RibD (R18, E42) and enolase (D87, R89) further stabilize the complex (Fig. 2D). The RibD homodimer is stabilized by its reductase domains, where two swapped β-sheets form the primary interaction interface (Fig. 2A). Each subunit contributes β-strands F150–M157 and E313–L318 that interact with I344–S347 and F334–S338 from the adjacent subunit (subunit B), creating two four-stranded β-sheets. The dimer is primarily held together by the extensive hydrogen-bonding network between the β-strand backbones, with additional stabilization from hydrophobic interactions, as β-strands I344–S347 and F334–S338 fit into a hydrophobic pocket in subunit A (Fig. 2B). In contrast, *E. coli* RibD dimerization involves two β-sheets (Stenmark et al., 2007) from subunits A and B without strand swapping (Fig. S3B and S3C). For a detailed comparison, see the Supplementary Text (Fig. S3A–D and Movie S1). The enolase dimer interface is conserved across species, with similar buried surface areas, contact shapes, and charge distributions (Kuhnel and Luisi, 2001). Dimerization is sensitive to ionic strength due to enriched charged residues at the interface (Kuhnel and Luisi, 2001). In this study, we show that the *Francisella* enolase dimer exhibits an extensive interaction interface, burying a solvent-inaccessible surface area of ∼ 3,200 Å^2^. Similar to *E. coli* enolase (Fig. S3E), the *Francisella* dimer interface is enriched with charged residues (Fig. S3F), but also incorporates hydrophobic interactions, highlighting species-specific stabilization differences.

**Figure 2.**
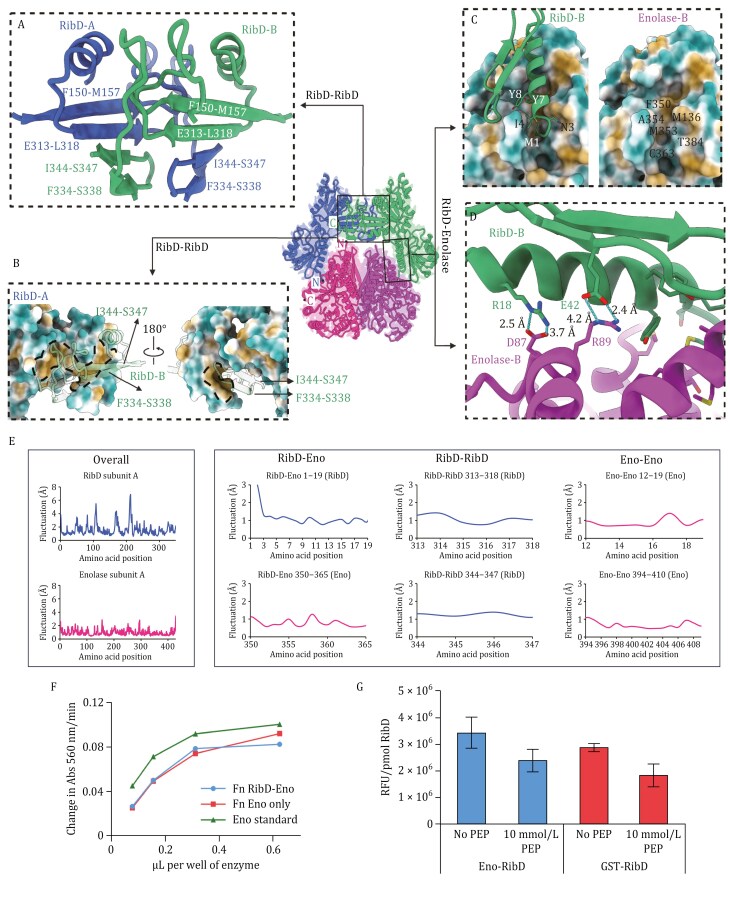
**Structural basis of complex assembly and enzymatic activities.** (A) Ribbon representation of interface between RibD-A and RibD-B. (B) Two views of surface representation with hydrophilic, neutral, and hydrophobic areas, indicated in cyan, white, and gold, respectively. The hydrophobic pocket is outlined by a dashed line. (C) Surface representation of interface between RibD and enolase with hydrophilic, neutral, and hydrophobic areas, indicated in cyan, white, and gold, respectively. (D) Charged interactions are shown between RibD and enolase with key residues labeled. (E) RMSF calculations of the three interfaces from MD simulations. Low RMSF (≤ 1.5 Å) values are consistently observed with interface-stabilizing residues. (F and G) Enzymatic assays show similar activities for individual proteins and the RibD-enolase complex. (F) Purified *F. novicida* RibD-Eno and enolase without RibD (from stocks of 2.6 and 2.4 µg/mL enolase) show similar levels of enolase enzymatic activity. (G) Quantification of the RibD reaction product by measurement of fluorescence with excitation at 408 nm and emission at 485 nm. Data indicate means ± standard errors of independent triplicate measurements of relative fluorescent units per pmol of RibD. GST, glutathione S-transferase.

To investigate the structural stability and dynamic behavior of the RibD-enolase complex in solution, we performed 300-nanosecond (ns) molecular dynamics simulations using GROMACS (Abraham et al., 2015). Structural fluctuations were assessed through Root Mean Square Fluctuation (RMSF) analysis, which quantifies the average deviation of each residue from its mean position throughout the simulation. Overall, RibD exhibited greater fluctuations than enolase (Fig. 2E), indicating higher flexibility, while enolase remained more rigid. Residues involved in complex formation displayed low fluctuations, suggesting strong and stable interface contacts (Fig. 2E). At the RibD-enolase interface, primarily involving RibD residues 1–8 and enolase residues 350–365, the average fluctuation was 1.10 Å. The RibD-RibD interface exhibited similar stability, while enolase’s homodimeric interface showed minimal fluctuations, suggesting that enolase may stabilize RibD in the complex. To gain insight into the mechanisms of catalysis, we further conducted computational docking and molecular dynamics simulations with ligands. These simulations revealed localized conformational changes within the substrate-binding sites and suggested a gating mechanism between RibD’s substrate and cofactor-binding sites to ensure efficient uptake and turnover (Figs. S4 and S5). For a detailed description of these experiments and results, see the Supplementary Text.

The interaction of *F. novicida* RibD and enolase raised the question as to whether this complex regulates their enzymatic activity. We found that enolase activity remained unchanged whether or not RibD is present (Fig. 2F). Additionally, adding RibD substrate [generated *in situ* by the addition of *E. coli* RibA (Fig. S6A) and GTP (guanosine-5'-triphosphate)] had no impact on enolase reaction rates for *F. novicida* enolase vs. the enolase-RibD complex (Fig. S6B). We next examined whether the addition of the enolase substrate, PEP, to the RibD-enolase complex alters the RibD enzymatic reaction rate compared with RibD without enolase. GST-tagged *Francisella* RibD was expressed, purified (Fig. S6C), and its enzymatic activity compared with RibD-enolase. Results showed similar activity for both, with PEP having the same effect on each (Figs. 2G, S6D and S6E). Thus, despite the physical association between RibD and enolase, enzymatic assays indicated that their catalytic activities are independent of each other. The complex may therefore have alternative functional roles that warrant further exploration. It is noteworthy that RibD and enolase are highly conserved across the *Francisella* genus, suggesting that the complex is likely present in other *Francisella* species, including the highly virulent *F. tularensis* subspecies. Future studies, including mutational analyses designed to disrupt the formation of the complex in conjunction with *in vivo* studies in eukaryotic host cells and in animals, may help elucidate the biological function of the complex.

The identification of the RibD-enolase complex was unexpected. The structural analysis, functional characterization, and molecular dynamics simulations of this complex reveal unique features of the *Francisella* complex, offering new insights into its metabolic organization and potential target for tularemia treatment. While we did not observe any evidence that the RibD-enolase complex formation mediates regulatory interaction between the two enzymatic pathways, it is possible that external factors other than the substrates that we examined (e.g., temperature or oxidative stress, or a more acidic pH) might enable such regulation. Given its role as a heat shock protein in yeast (Iida and Yahara, 1985) and a hypoxic stress response protein in mammalian cells (Aaronson et al., 1995), *Francisella* enolase might act as a chaperone to protect and stabilize RibD during stresses encountered in the host cell environment. Broadly, this serendipitous discovery of the *Francisella* RibD-enolase complex, akin to the cryoID approach for determining and identifying structures from crude cellular isolates (Ho et al., 2020), highlights the power of high-resolution cryoEM to uncover unforeseen biological relationships.

## Supplementary Material

pwaf045_Supplementary_Data

## Footnotes

We thank Xian Xia for assistance in cryoEM imaging and Kaelyn Y. Feng for editorial assistance.

This work was supported by NIH grants (R01AI151055 to MAH and ZHZ, and R01GM071940 to ZHZ). We acknowledge the use of resources at the Electron Imaging Center for NanoSystems supported by US NIH (1S10OD018111) and the US National Science Foundation (DBI-1338135 and DMR-1548924).

The authors declare no competing interests. All the authors declare their agreement to publish.

The cryo-EM density map has been deposited in the Electron Microscopy Data Bank under accession codes EMD-49592. The atomic coordinate has been deposited in the Protein Data Bank under accession codes 9NO2.

Z.H.Z. and M.A.H. supervised the project. D.L.C. and B.-Y.L. prepared protein sample; X.L. made cryoEM grids, performed cryoEM imaging, and data processing; X.L. built the atomic model and illustrated the structures; D.L.C. and B.-Y.L. prepared bacterial strains, performed biochemistry analysis, enzyme activity assays, and bacterial two-hybrid assay. R.A. performed molecular dynamics simulation and analyzed the results. X.L., D.L.C., B.-Y.L., R.A., M.A.H. and Z.H.Z. interpreted the data and wrote the manuscript; and all authors reviewed and approved the paper.
