# Supplementary material for: Structure, identification, and characterization of the RibD-enolase complex in Francisella
Source: Protein Cell. 2025 Jun 5;16(12):1048–53. doi: 10.1093/procel/pwaf045 (PMC12742849; doi:10.1093/procel/pwaf045)
Supplement: pwaf045_Supplementary_Data [file pwaf045_supplementary_data.zip › pwaf045_suppl_Supplementary_Figures_S1-S6.pdf]

Supplemental Information

Title: Structure, identification and characterization of the RibD-enolase complex in *Francisella*

Xiaoyu Liu[1,2], Daniel L. Clemens[3], Bai-Yu Lee[3], Roman Aguirre[1,2,4], Marcus A. Horwitz[1,3,*], Z. Hong Zhou[1,2,4,*]

[1]Department of Microbiology, Immunology and Molecular Genetics, University of California, Los Angeles (UCLA), Los Angeles, CA 90095, USA;

[2]The California NanoSystems Institute (CNSI), UCLA, Los Angeles, CA 90095, USA;

[3]Department of Medicine, UCLA, Los Angeles, CA 90095, USA;

[4]Department of Chemistry and Biochemistry, University of California, Los Angeles (UCLA), Los Angeles, CA 90095, USA;

*Correspondence: MHorwitz@mednet.ucla.edu (infectious diseases and biochemistry) or Hong.Zhou@ucla.edu (cryoEM and structures)

Running head (50 chars): RibD and enolase form a complex in *Francisella*

Keywords: *Francisella*, cryoEM, RibD-enolase, enzyme complex

Methods

Purification of protein sample

Francisella novicida expressing FLAG epitope tagged PdpC was grown in trypticase soy broth with 0.2% cysteine (TSBC), 5% KCl, 0.1 mg/mL $FeSO_4$, and 5 mM betaine to an optical density (540 nm) of 2.0. Bacteria were pelleted by centrifugation (4000 g for 90 min at 4°C), resuspended in 50 mM sodium phosphate, pH 7.4, 1 mM EDTA, 1 mM PMSF, 1 mM NEM, and 1:100 protease inhibitor cocktail (HY-K0010, MedChemExpress), and lysozyme (1 mg/mL) and disrupted by sonication on ice with a probe tip sonicator. The sonicate was clarified by ultracentrifugation (44,400 g for 90 min at 4°C) and NaCl and Mega-9 detergent added to achieve concentrations of 0.15 M and 0.2%, respectively. The supernate was rotated overnight with 0.5 mL of anti-FLAG M2-agarose (Sigma-Aldrich), washed extensively with 50 mM sodium phosphate with 0.15 M NaCl, 1 mM EDTA, and eluted with 3X-FLAG peptide (0.1 mg/mL in the same buffer). The eluate was concentrated with a 100 kDa MWCO filter (Amicon Ultra 15) and used for cryoEM.

Generation of epitope tagged strains of *F. novicida*

PCR products of the 1.3 kb enolase gene with a C-terminal 3xFLAG tag and 1.2 kb of its downstream genomic sequence, including a repeat of the last 23 nucleotides of the enolase (FTN_0621) gene to preserve the ribosomal binding site, were ligated into pJC84 (Wehrly et al., 2009). Similarly, the 1.1 kb *rib*D gene (FTN_0114) with a C-terminal ALFA tag and 0.3 kb of its upstream region and 1.2 kb of its downstream genomic region, including a repeat of the last 24 nucleotides of the *rib*D gene, were cloned into pJC84. Plasmid constructs were confirmed by nucleotide sequencing (Plasmidsaurus) and used for transformation of *F. novicida* U112. Transformants grown on trypticase soy agar containing 0.1% cysteine (TSAC) and 20 µg/ml kanamycin were screened using colony PCR and subsequently counterselected on TSAC containing 7% sucrose. Replacement of the wildtype copy of enolase or *rib*D gene with the

epitope tagged gene was validated by Western blotting using FLAG-HRP (Sigma-Aldrich, 1:30000) or ALFA-HRP (Synaptic Systems, 1:4000) antibodies.

Affinity pull-down of RibD-ALFA

F. novicida expressing enolase-FLAG with or without co-expression of RibD-ALFA were grown in 200 mL of TSBC to an optical density (540 nm) of 2, pelleted by centrifugation, resuspended in 50 mM Tris HCl, pH 8, 0.1 M NaCl, 1 mg/mL Lysozyme, 1 mM PMSF, 2 mM NEM, 1:100 protease inhibitor cocktail (MedChemExpress), and 0.2% Tween-20, and sonicated in an ice bath with a probe tip sonicator. The sonicated pellets were clarified by ultracentrifugation (44,400 g for 90 min at 4°C) and the supernates were each incubated with 0.05 mL of ALFA Selector Resin CE (Synaptic Systems) overnight at 4°C with end-over-end rotation, washed with 50 mM Tris HCl, pH 8, 0.1 M NaCl, and eluted in this buffer with 0.2 mM ALFA peptide.

Affinity pull-down of enolase-FLAG

The clarified lysate of *F. novicida* expressing RibD-ALFA and enolase-FLAG which did not bind to the ALFA-selector resin described above was incubated at 4°C overnight while rotating with 1.5 mL of M2-anti-FLAG agarose (Sigma Aldrich), washed with 50 mM Tris HCl, pH 8, 0.1 M NaCl, and eluted in this buffer with 0.1 mg/mL 3x-FLAG-peptide.

Western Immunoblotting

Proteins were separated on Any kD Mini-Protean TGX Stain-free gels (Bio-Rad) and visualized using ChemiDoc Imaging System (Bio-Rad) prior to transblotting onto a 0.2 μm nitrocellulose membrane. The membrane blot was blocked using EveryBlock blocking buffer and probed with HRP-conjugated camelid single domain antibody to ALFA epitope tag (Synaptic Systems) or HRP-conjugated M2 mouse monoclonal anti-FLAG epitope tag (Sigma Aldrich) at a dilution of 1:4000 or 1:30000, respectively. Chemiluminescent signals were developed by incubating with Clarity Western ECL substrates (Bio-Rad) and detected using the ChemiDoc Imaging System.

Cloning and recombinant expression of RibA and RibD

The full-length gene encoding *rib*A was amplified from genomic DNA of *E. coli* NEB5-alpha, digested with *Nde*I and *Xho*I and ligated into expression vector pET28b (Novagen) downstream of a hexahistidine tag and a thrombin cleavage site. The *rib*D gene was amplified from *F. novicida* U112 genomic DNA, digested with *Bam*HI and *Xho*I, and cloned into the vector pGEX-4T-1 for expressing GST-RibD fusion protein with a thrombin cleavage site. The expression plasmids were confirmed by nucleotide sequencing and transformed into *E. coli* BL21(DE3) strain (Novagen). For production of the recombinant proteins, BL21(DE3) cells harboring the pET28a or pGEX-4T-1 construct were grown at 37°C in LB medium until the optical density at 600 nm reached 0.6-0.8 at which time IPTG was added to achieve a final concentration of 0.5 or 1 mM. The cultures were grown at 18°C for 16-20 h and harvested by centrifugation at 3500 g for 20 min and stored at -80°C.

Purification of *E. coli* histidine-tagged RibA

Bacterial pellets of *E. coli* expressing histidine-tagged RibA were resuspended in 50 mM sodium phosphate, pH 8.0, 0.3 M NaCl, 10 mM imidazole, 1 mM PMSF, and EDTA-free protease inhibitor cocktail (MedChemExpress), sonicated with a probe tip sonicator, and clarified by ultracentrifugation. The clarified supernate was rotated overnight at 4°C with 0.5 mL of Ni-NTA-agarose (Pierce), washed extensively with the lysis buffer and lysis buffer containing 30 mM imidazole and eluted with lysis buffer containing 0.3 M imidazole. Fractions containing RibA were desalted with an Excellulose desalting column equilibrated with 0.1 M Tris HCl, pH 8.

Purification of GST-tagged *F. novicida* RibD

Pellets of *E. coli* expressing GST-RibD grown as described above were thawed; resuspended in PBS (140 mM NaCl, 2.7 mM KCl, 10 mM Na_2HPO_4, 1.8 mM KH_2PO4, pH 7.3) with 1% TX-100, 1 mM DTT, and 1:100 protease inhibitor cocktail (MedChemExpress); sonicated on ice with a

probe tip sonicator; clarified by ultracentrifugation; and incubated with 1 mL of Glutathione-Sepharose 4B (Cytiva Life Sciences) overnight at 4°C while rotating end-over-end. The resin was washed sequentially with PBS with 1% TX-100, and 50 mM Tris, 150 mM NaCl, pH 8.0, and eluted with 10 mM reduced glutathione in 50 mM Tris, 150 mM NaCl, pH 8.0.

Enzyme activity assays

Enolase enzyme activity was measured by a colorimetric (570 nm) assay using the Enolase Activity Assay Kit (MAK178−1KT, Sigma-Aldrich, St. Louis, MO, USA), which follows the conversion of D-2-phosphoglycerate to PEP by measuring the formation of an intermediate that reacts with a peroxidase substrate.

RibD deaminase and reductase activities were measured at 37°C by a modification of published methods (Richter et al., 1997; Graupner et al., 2002). The RibD enzyme assay reaction contained 50 mM Tris, 150 mM NaCl, pH 8.0, *E. coli* his-tagged RibA (0.17 mg/mL), 10 mM GTP, 10 mM NADPH, 10 mM $MgCl_2$, 10 mM DTT, 10 mM glucose-6-phosphate and 1 U/mL glucose-6-phosphate dehydrogenase (Catalog G5885, Sigma Aldrich) for NADPH regeneration. In this system, RibD substrate (2,5-diamino-6-ribosylamino-4(3H)-pyrimidone 5' phosphate) is generated in situ from GTP by *E. coli* RibA. The RibD deaminase and reductase reactions lead to generation of the reaction product 5-amino-6-ribitylamino-2,4(1H,3H)-pyrimidinedione 5'-phosphate. The reaction is stopped by addition of methanolic diacetyl and boiling for 30 min, which derivatizes the substrate to a blue fluorescent product, 6,7-dimethylpterin, and the reaction product to a green fluorescent product, 6,7-dimethyl-8-ribityllumazine 5'-phosphate. Denatured protein is removed by centrifugation at 14,000 g for 30 min, supernatants are evaporated to dryness under vacuum, redissolved in water, spotted onto C-18-W Silica TLC plates (Sorbtech, Norcross, GA) and developed in 20 mM sodium acetate, pH 6, 8% methanol. The blue substrate and green reaction product bands were imaged under long wavelength UV light and areas of the TLC plate corresponding to the green bands were scraped, eluted into 20

mM sodium acetate, pH 6, 80% methanol, and fluorescence measured with a Tecan Spectramax fluorimeter with excitation 408 nm and emission 485 nm.

Bacterial two-hybrid (BACTH) assay

The genes encoding enolase and RibD were amplified from *F. novicida* U112 genomic DNA or from synthetic gene fragments codon optimized for *E. coli* (Twist Bioscience) and cloned into BACTH expression plasmids (Euromedex) fusing with adenylate cyclase T18-fragment (pUT18 or pUT18c, ampicillin-resistant) and T25-fragment (pKT25 or pKNT25, kanamycin-resistant). The expression constructs were transformed into *E. coli* NEB5-alpha F' I^q (NEB) and verified by restriction digestions and nucleotide sequencing. Two compatible plasmids, expressing the T18 and T25 fusion, respectively, were co-transformed into *E. coli* strain BTH101 by electroporation, and the transformants were selected on LB agar containing 50 µg/ml kanamycin and 100 µg/ml carbenicillin. For detection of protein interactions, antibiotic resistant clones were grown in LB medium containing 1 mM IPTG at 37°C for 16 h, and 3 µl of the cultures were spotted onto LB agar containing 1 mM IPTG and 40 µg/ml X-gal. The agar plates were incubated at 30°C for 48h.

Graphene grids preparation

The single layer graphene on Cu foil was purchased from Graphene Supermarket. Graphene grid fabrication was done similarly as described in previous work (Han et al., 2020). Briefly, MMA EL 6 was used to coat the graphene on copper foil with a spin coater at ~2500 rpm for 1 min. The graphene on the backside of the copper foil was removed by glow-discharge. Then the copper foil of the MMA/graphene/Cu was dissolved using 1 M ammonium persulfate (APS). The remaining film was transferred to deionized (DI) water and held for 20 min. We used Quantifoil 1.2/1.3 holy girds to harvest the MMA/graphene film and baked the grids for 20 min at 130 °C. The grids were then placed in acetone to dissolve the MMA, followed by transferring grids to isopropyl alcohol (IPA) to remove the acetone residue, and baked for another 20 min to dry. A

UV/ozone cleaner was used for 10 min to render graphene grids hydrophilic. The graphene grids were used on the same day they were prepared.

CryoEM sample preparation and image collection

For cryoEM sample preparation, an aliquot of 3 μL of purified protein sample was applied to prepared graphene grids. The grid was blotted with filter paper (Ted Pella) and then flash-frozen in liquid ethane with an FEI Vitrobot Mark IV (Thermo Fisher Scientific). Freezing conditions were optimized by checking the grids with an FEI TF20 cryoEM instrument. The best grids with optimal ice thickness and particle distribution were obtained with the Vitrobot settings of 8 °C temperature, 100% humidity, 30 s waiting time, 8 s blotting time and 8 blotting force. The prepared cryoEM grids were loaded into a Titan Krios electron microscope (Thermo Fisher Scientific) with a Gatan imaging filter (GIF) Quantum LS and a K3 Summit direct electron detector. The microscope was operated at 300 kV with the GIF energy-filtering slit width setting to 20 eV in super-resolution mode. Movies were collected with SerialEM (Mastronarde, 2005) at a pixel size of 1.1 Å on the sample level with a total dosage of ~50 e–/Å2/movie. 39,482 movies were collected.

Single particle cryoEM reconstruction and model building

The single particle analysis workflow is outlined in Supplementary Fig. S1. Motion correction and defocus value determination were performed in Live CryoSPARC (Punjani et al., 2017). The following data processing was performed with CryoSPARC. 17,070,858 particles were picked by Topaz (Bepler et al., 2019), extracted with boxsize of 300 × 300 pixels and binned 2× to 150 × 150 pixels (2.2 Å per pixel) to speed up the data processing procedure. 407,158 particles were selected from 2D classification. After ab initio reconstruction and homo-refinement, the particles were re-extracted with a box size of 300 × 300 pixels (1.1 Å per pixel). Local CTF refinement, non-uniform refinement with C2 symmetry, and 3D classification were then performed. A total of 356,717 particles from two classes of 3D classification were used for non-uniform refinement,

resulting in a final reconstruction at 3.05 Å resolution. The resolution of the map was estimated from the gold-standard Fourier shell correlation criterion, FSC = 0.143. Data collection and processing statistics are summarized in Table S1.

The reconstructed cryoEM map was used to generate a 3D model trace by DeepTracer (Pfab et al., 2021). Based on the sequence model provided by DeepTracer, a BLAST analysis against the *Francisella novicida* protein database in Uniprot identified the proteins as RibD and enolase. Model building is started with the model generated by DeepTracer. The atomic model of RibD-enolase complex was built and refined manually in COOT (Emsley and Cowtan, 2004) and further refined in Phenix (Afonine et al., 2018). Refinement statistics of the model are summarized in Table S1. Visualization of the atomic model, including figures and movies, was accomplished in UCSF ChimeraX (Pettersen et al., 2021).

Molecular dynamics simulation and ligand docking

Both apo and holo RibD-enolase files were prepared using the CHARMM-GUI webserver solution builder tool (Jo et al., 2008) with the AMBER36 force field at 298K and a pH of 7.5, outputting GROMACS-compatible files. The solvated file was then imported into GROMACS (Abraham et al., 2015) and subjected to pressure and temperature equilibrations for 100 picosecond (ps) each. After verification of proper equilibration, the protein complex was then simulated for 300 nanoseconds (ns). All simulations were run locally using an Nvidia RTX 3070 Ti GPU. The trajectory was then made whole and unwrapped, allowing for direct visualization and analysis using the built-in gromacs tools for RMSF and RMSD calculations. XVG files were manipulated using Microsoft Excel.

For the holo simulation, Chai-1 (Chai Discovery Team et al., 2024) was used to predict the substrate and ion-binding pockets within the RibD-enolase complex. Afterwards, the experimentally-determined model was overlaid into the predicted complex:ligand structure using

chimeraX (Pettersen et al., 2021). The predicted structure was then removed, resulting in the ligands being docked into the experimentally determined model. Using the ligand reader function in CHARMM-GUI (Jo et al., 2008), each individual substrate was extracted and and placed into the CGENFF server, assigning force fields to the molecules. Finally, the mol2 output files were used in tandem with the Eno/RibD pdb files in CHARMM-GUI's solution builder, generating a simulation-ready folder.

Supplementary Text

Comparison of RibD and enolase between *Francisella* and *E. coli* reveals diversification

The structural and biochemical data in this study establish that RibD and enolase form a stable complex in *Francisella*. In contrast, in *E. coli*, RibD and enolase exist independently, with their structures resolved separately (Kuhnel and Luisi, 2001; Stenmark et al., 2007). To gain insights into the structural differences, we compared the complexed protein structures from *Francisella* with the individually resolved structures from *E. coli*. RibD from *Francisella* and *E. coli* share a majority of their secondary structural elements (Fig. S3D). Structural alignment of RibD from *Francisella* and *E. coli* along their symmetry axis revealed a notable difference: the protomers in *Francisella* are positioned further apart than those in *E. coli* (Fig S3A and Movie S1). The wider space between the protomers may facilitate the binding of enolase. Zoomed-in views provide a closer look at the variations observed at the dimerization interface between the two species. In *Francisella*, as described above, the dimerization interface of RibD is predominantly stabilized by swapped β-strands contributed by two protomers (Fig. S3B). In contrast, the dimerization interface in *E. coli* RibD is formed by two β-sheets (Stenmark et al., 2007), contributed by subunits A and B, without strand swapping (Fig. S3C). However, we have found that AlphaFold does not yield reliable predictions of RibD β-strand swapping. Therefore, experimental

observations, rather than *in silico* analyses, are required to ascertain whether β-strand swapping and RibD-enolase complex formation occur in other species and to gain insights into the evolution of these features. The comparison of enolase structures from *Francisella* and *E. coli* (PDB: 1E9I) (Kuhnel and Luisi, 2001) reveals a high degree of similarity, with a root mean square deviation (RMSD) of 2.0 Å across all 423 pairs (Fig. S4E), supporting the understanding that enolases are highly conserved enzymes among bacteria. These structural variations likely reflect functional adaptions specific to the metabolic or regulatory needs of each species.

Molecular dynamics simulations show substrate binding and substrate-cofactor occlusion

To gain insight into mechanisms of catalysis, we carried out computational docking and molecular dynamics simulation. Chai-1 (Chai Discovery Team et al., 2024) was used to dock DARPP, ARPP, NADPH and Zn^{2+} to RibD subunits and 2-phospho-D-glycerate (PGA or 2PG) and Mg^{2+} to enolase subunits, resulting in 12 total docked ligands (Fig. S4). Afterwards, the experimentally determined structure was overlayed onto Chai-1's predicted ligands, and the predicted RibD-enolase complex was removed. The protein-ligand complex was then simulated using GROMACS (Abraham et al., 2015) for 300 ns. The RMSF values between the apo and holo simulations were compared to observe any substantial changes between amino acids at their respective binding sites.

The reductase domain of RibD subunit A had ARPP bound throughout the entirety of the 300 ns, but experienced significant local conformational changes at loop 163-174 to better accommodate ARPP within the substrate-binding pocket (Fig. S4A). This loop, containing a crucial D167, clamps down to interact with ARPP (Fig. S4B). On RibD subunit B, NADPH remained bound throughout the entire simulation. There was no noticeable difference between the apo and holo binding sites, with only minor translations from global RibD movements (Fig. S4A). Within the deaminase domain of both subunits, the substrates remained bound

throughout the entire simulation (Fig. S4A, S5C), showing large fluctuation differences between the apo and holo forms at residues H52 and K64. While DARPP only undergoes small translational changes, the aforementioned key residues in the deaminase domain have drastic fluctuation changes.

Surprisingly, the simulation trajectories of the holo reductase domain of RibD, containing both ARPP and NADPH, indicated that each subunit only binds one molecule at a time. When NADPH was lodged in one subunit, ARPP would be released, and vice versa (Fig. S4B). This dislodgement is due to the occlusion caused by the highly flexible loop 163-174, which stabilizes components of both active sites. It cannot accommodate both at the same time, leading to dislodgement of the unattended molecule, but at different time scales (Fig S5A, S5B). This sheds light into the mechanistic approach for the turnover of fully-realized molecules, allowing for new substrates and reduced NADPH to bind to RibD.

Comparisons between apo and holo simulations of the enolase-2PG binding site do not show any noticeable conformational changes (Fig. S4C). Particularly, holo Asp314, responsible for coordinating with the Mg^{2+} ion, does not exhibit a large mean fluctuation in contrast to its apo form. Both 2PG molecules do not undergo any large conformational changes (Fig. S5D), other than minor translations from their original locations. This relatively low change in both RMSD and RMSF values suggests no substantial conformational differences between enolase's apo and holo forms. These observations show that enolase lacks flexibility or dynamic capabilities in its substrate-binding pocket, resulting in a small and static active site.

Molecular dynamics simulations of the holo RibD-enolase complex suggest a gating mechanism within the broader ARPP/NAPDH binding region, resulting in the occlusion and eventual turnover of both the ligand and cofactor. This dynamic behavior aligns with the previous structural studies of RibD from *E. coli*, which proposed similar conformational transitions (Stenmark et al., 2007). The *E. coli* RibD structures captured the loop in the reductase active site in different states: accessible, substrate occluded, and fully occluded with

both the cofactor and substrate (Stenmark et al., 2007). By integrating our findings with these prior observations, we can corroborate the existence of some of these catalytically viable states, namely the accessible states, in which NADPH and ARPP are able to bind, but then transitions into the dislodging of NADPH (cofactor occluding state), potentially turning over its life cycle due to being oxidized. The other simulated holo RibD subunit keeps the flexible active site loop close to NADPH to stabilize it, akin to the substrate occluded state, but does not allow for proper ARPP binding, resulting in the reductase substrate's departure from the complex. This simulation result sheds insight into this dynamic region of the RibD substrate, which requires careful modulation of both its substrate and cofactor to ensure efficient uptake and turnover of each.

Table S1. CryoEM data collection, refinement and validation statistics

	**RibD-enolase EMDB 49592 PDB 9NO2**
**Data collection and processing**	
Magnification	81000
Voltage (kV)	300
Electron exposure ($e^-/Å^2$)	50
Defocus range (μm)	-1.8 to -2.6
Pixel size (Å)	1.1
Symmetry imposed	C2
Particle number	356717
Map resolution	3.05
FSC threshold	0.143
**Refinement**	
Map sharpening *B* factor ($Å^2$)	-155
Model composition	
Non-hydrogen atoms	11996
Protein residues	1556
Ligand	
B factors ($Å^2$)	
Protein	33.6
Ligand	
R.m.s. deviations	
Bond lengths (Å)	0.003
Bond angles (°)	0.622
Validation	
MolProbity score	1.62
Clashscore	4.67
Poor rotamers (%)	0.00
Ramachandran plot	
Favored (%)	94.38
Allowed (%)	5.62
Disallowed (%)	0.00

Supplementary Figures

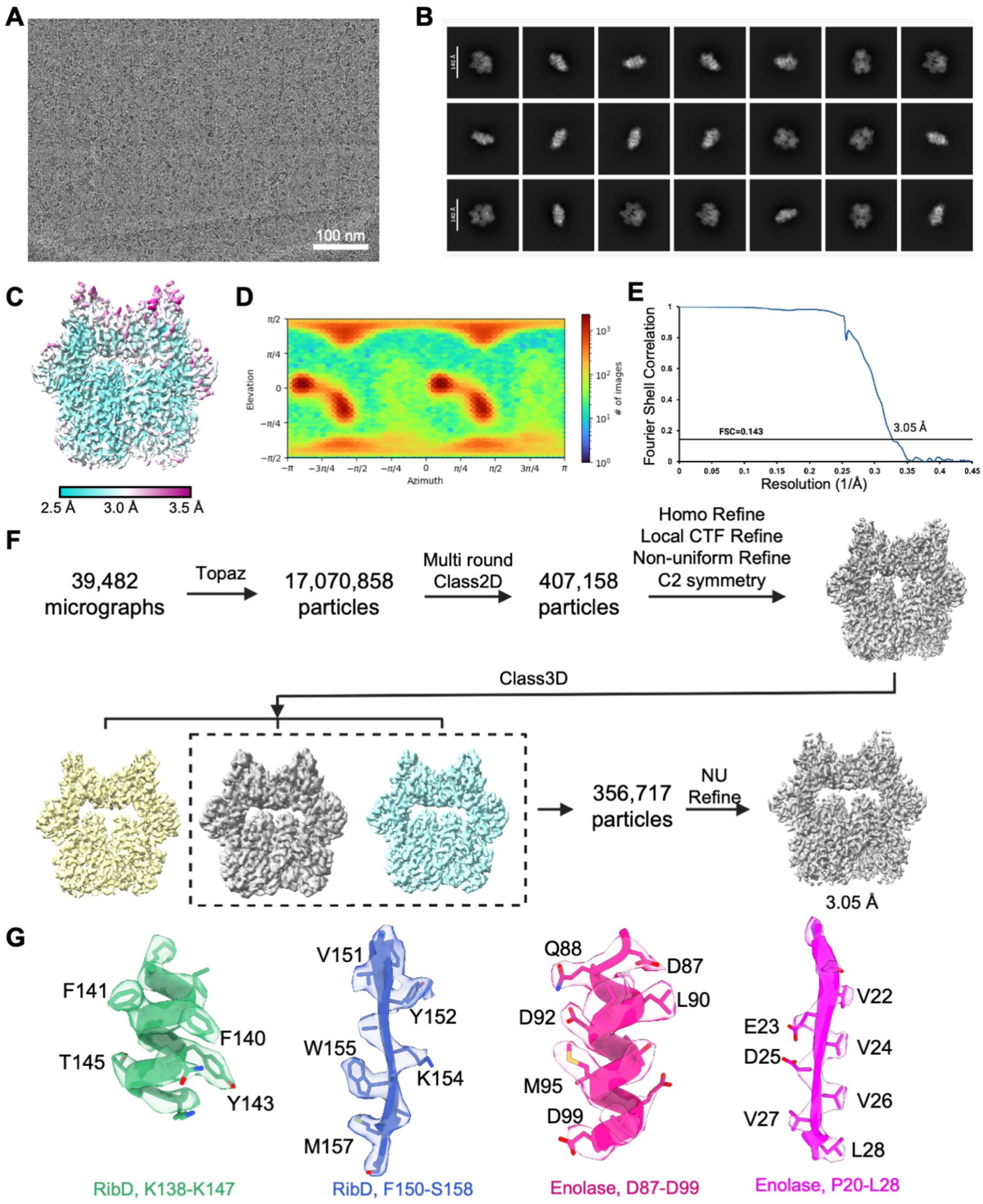

Figure S1. Data processing flowchart. A. Motion-corrected cryoEM micrograph. **B.** Representative 2D class averages of RibD-enolase particles. **C.** CryoEM map colored with local resolution. **D.** The angular distribution of the particles for final 3D reconstruction. **E.** Gold-standard Fourier shell correlation (FSC) curve for the cryoEM map. **F.** Flow chart of cryoEM data processing. **G.** Local cryoEM density maps.

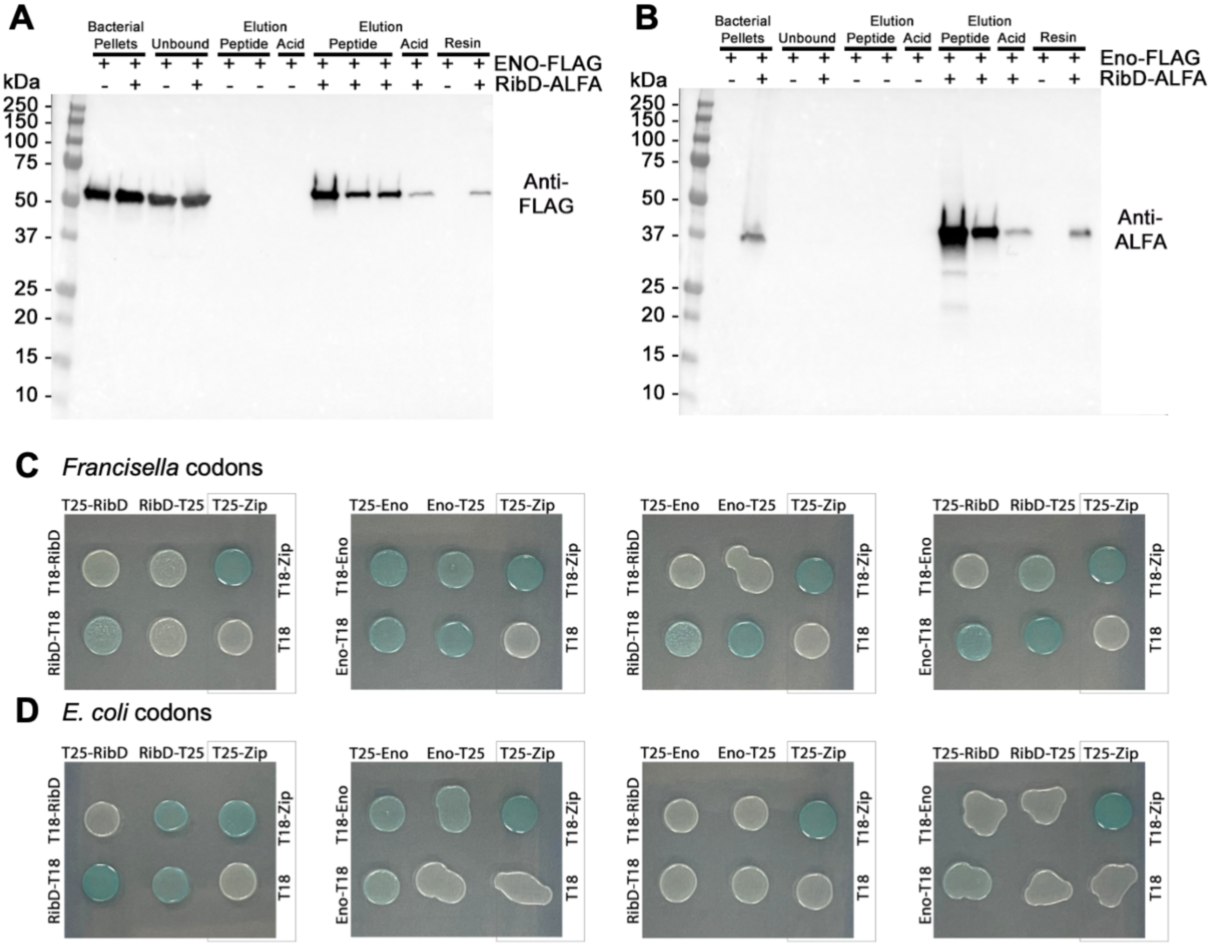

Figure S2. RibD and enolase form a complex in vivo. A-B. Western blot analysis of pull-down samples in Fig. 1E (*F. novicida* strains expressing Enolase-FLAG with or without RibD-ALFA). **A.** Anti-FLAG Western Immunoblot confirms that Enolase-FLAG is pulled down by anti-ALFA resin in the strain that co-expresses RibD-ALFA and Enolase-FLAG. It is not pulled down in the control strain that expresses Enolase-FLAG and untagged RibD. **B.** Western Immunoblot is repeated to immunostain for ALFA-tagged RibD. **C-D.** Bacterial two-hybrid assay detects RibD-RibD and enolase-enolase self-interactions and the interactions between RibD and enolase. *F. novicida* RibD and enolase encoded using native codons **(C)** or *E. coli* codons **(D)** were expressed as fusions with T18 or T25 catalytic domains of *Bordetella pertussis* adenylate cyclase in *E. coli* BTH101 with IPTG induction. Bacteria were spotted onto LB agar containing X-gal, which acted as a visual indicator to detect protein-protein interactions with blue colonies indicating a positive interaction. Pairwise interactions of RibD and enolase were labeled on the top and left of each spot. Interaction of T18-Zip and T25-Zip served as the positive control, whereas interaction of T18 and T25-Zip served as the negative control.

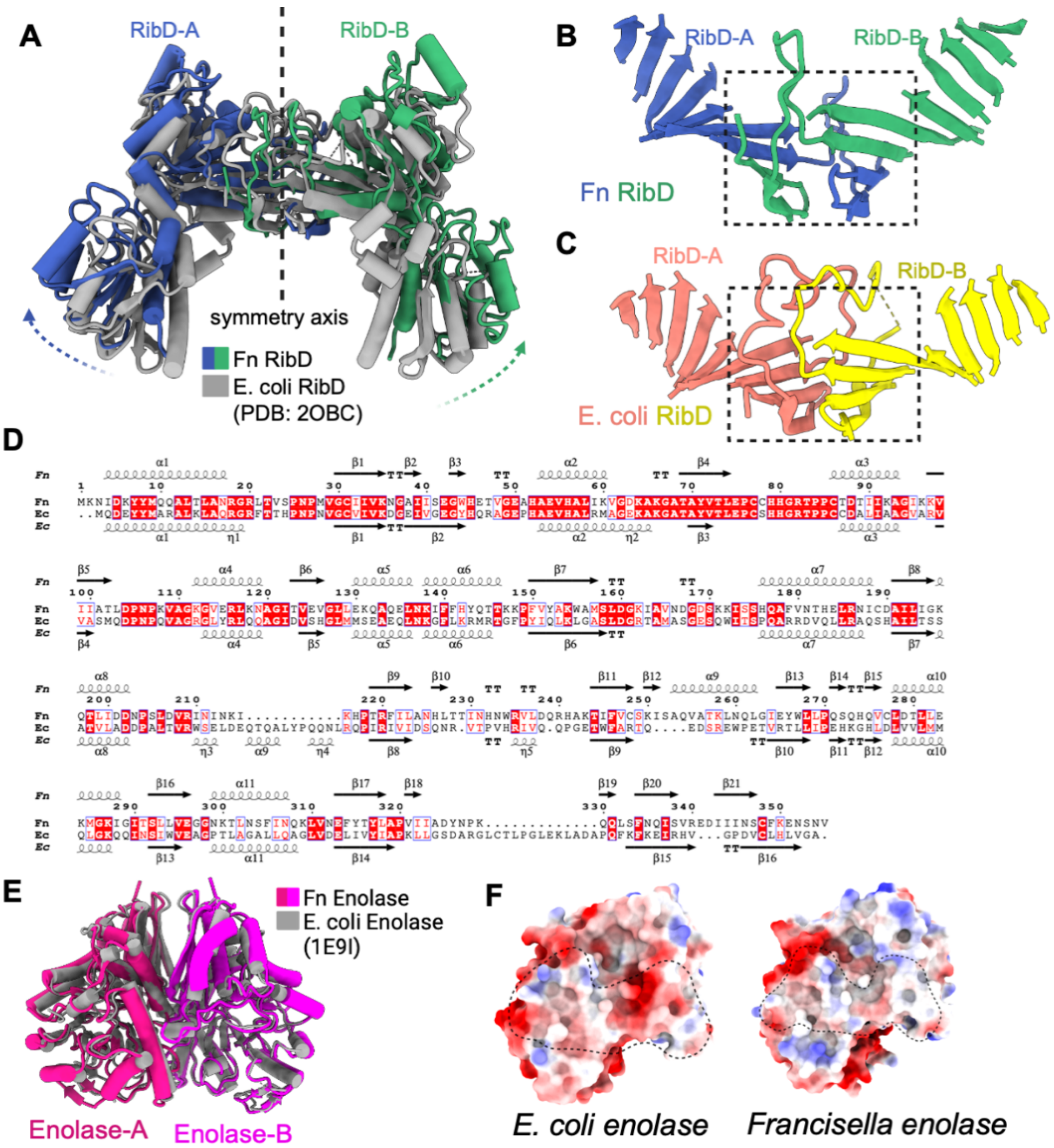

Figure S3. Comparison of RibD and enolase between *Francisella* (Fn) and *E. coli*. A. Alignment of Fn RibD and *E. coli* RibD. Fn RibD is colored as blue and green, *E. coli* RibD is colored as gray. The dashed arrows indicate the changes from *E. coli* RibD to Fn RibD. **B.** Ribbon representation of Fn RibD showing the swapped β-strands between RibD-A and RibD-B. **C.** Ribbon representation of *E. coli* RibD showing the interface between RibD-A and RibD-B. **D.** Sequence alignment of *F. novicida* RibD and *E. coli* RibD. The sequence alignment was done with Clustal Omega and displayed with ESPript 3. Secondary structures of Fn RibD determined in this study are shown on top. Secondary structures labeled at the bottom were done according to *E. coli* structural superposition (PDB accession no. 2OBC). **E.** Structure alignment of Fn Enolase and *E. coli* Enolase showing that the architectures are similar. **F.** Electrostatic surface potential of enolase dimer interfaces with positive, neutral, and negative electrostatic potentials indicated in blue, white, and red, respectively. The left panel shows *E. coli* enolase (PDB 1E9I) and the right one shows *Francisella* enolase from this study. The interface area is outlined with a dashed line. The dimer interface is enriched in charged residues.

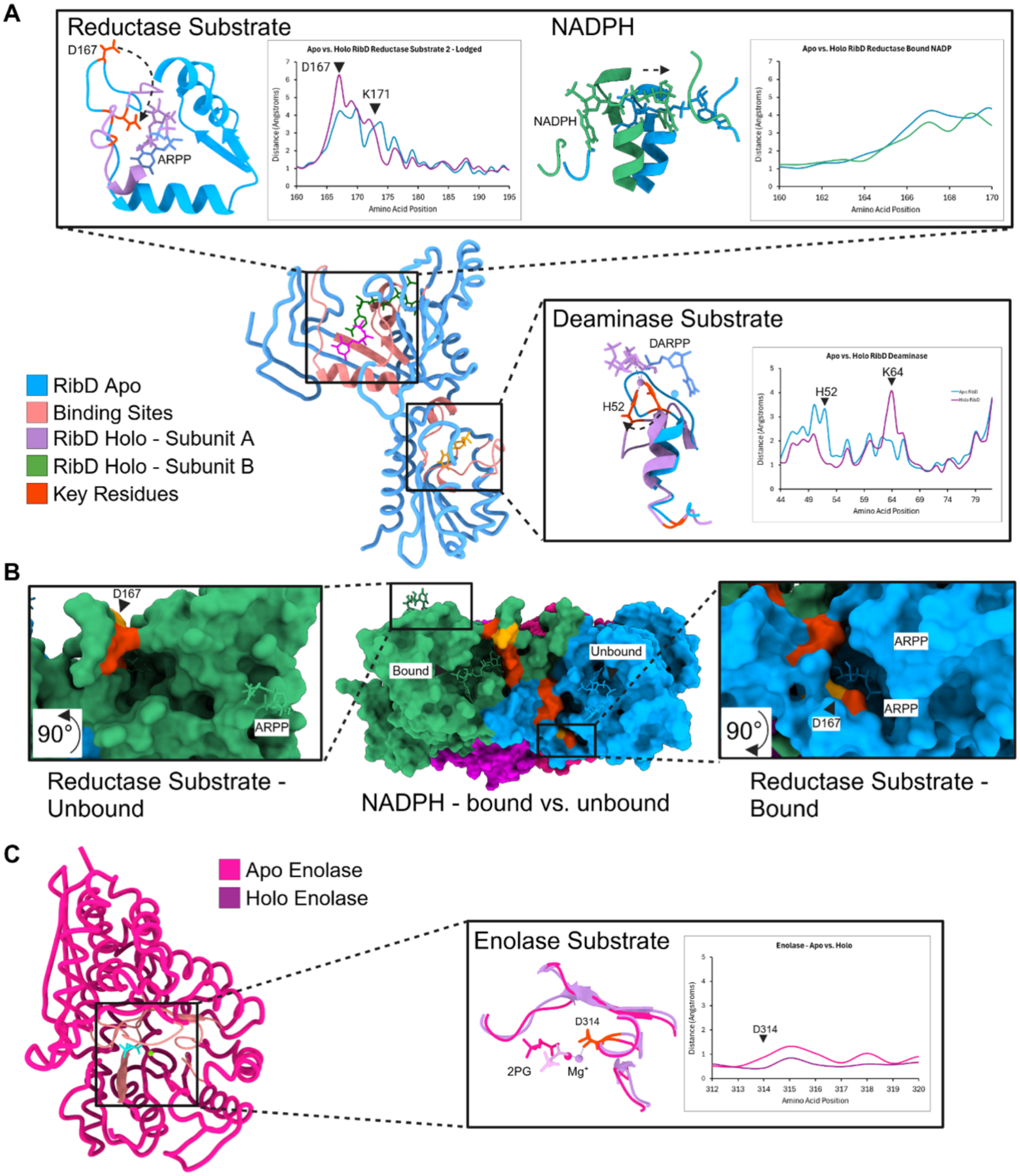

Figure S4. MD simulations RMSF comparisons between apo and holo forms. A. Active site fluctuations and local movement to accommodate substrate reductase domain loop 163-174 experiences a large conformational change to bind ARPP, resulting in D167 clamping onto the molecule. However, bound NADPH did not experience large local fluctuations at its binding site. **B.** RibD subunit 1 (blue) shows a partially dislodged NADPH due to the binding site rearrangement from the drastic movement of D167 to the interior of the Reductase substrate binding site to stabilize the substrate (right-hand inset). Conversely, RibD subunit 2 (green) shows a bound NADPH with key residues providing shape complementarity at its active site. This results in the dislodging of the reductase substrate from RibD. **C.** Both Enolase subunits bound their substrate in a rigid manner through key residue D314, showing no drastic RMSF changes between its apo and holo forms.

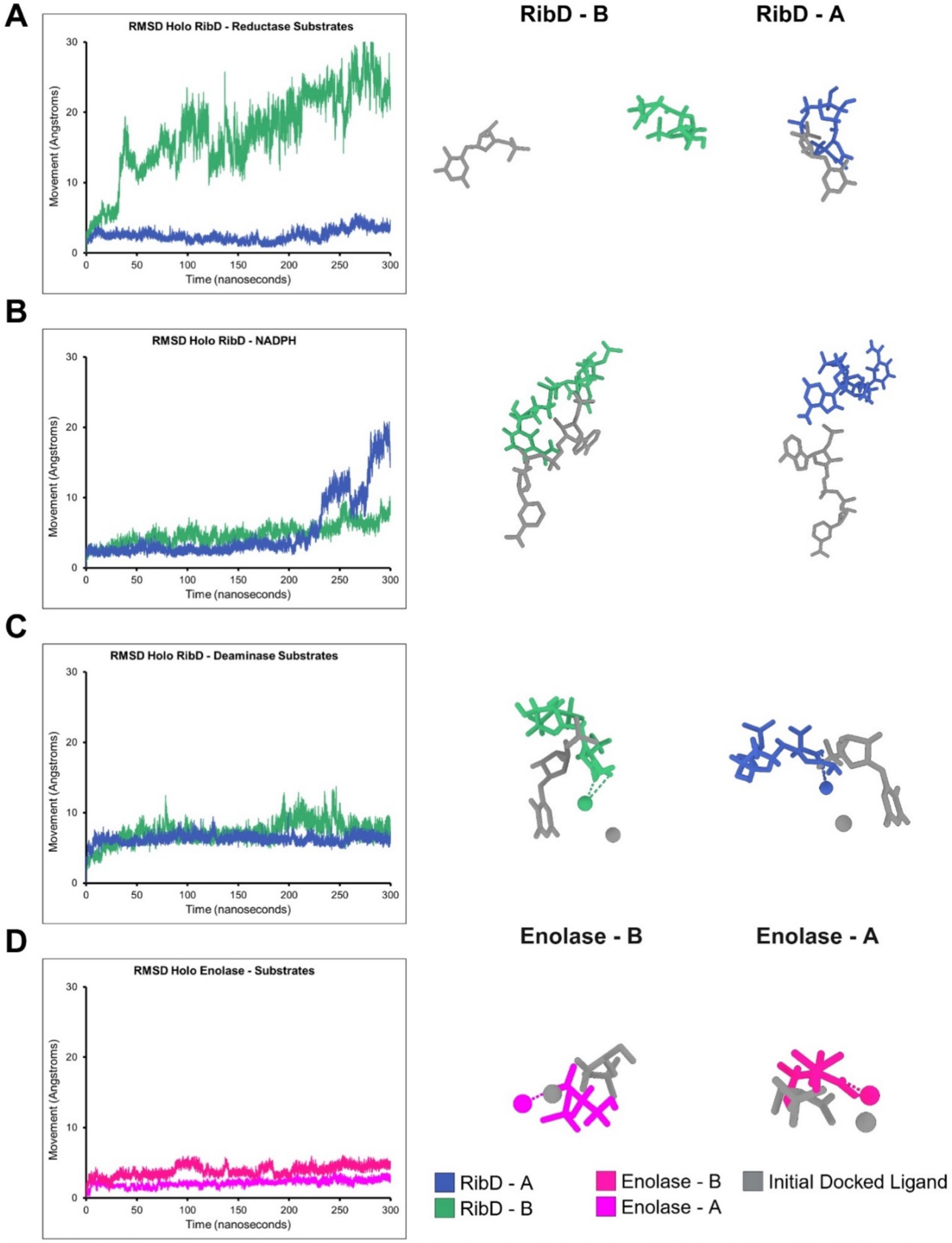

Fig S5. Root Mean Square Deviation (RMSD) plots of each substrate from MD simulations. RMSD plots the deviation of the holo RibD-enolase complex from the superimposed experimental structure throughout the 300 ns. This is shown for: **A** The reductase substrates; **B** NADPH cofactors; **C** Deaminase substrates; and **D** enolase substrates. The right-hand panels show the distance travel and conformational change of the simulated molecules (color) in comparison to the initial docked molecules (gray).

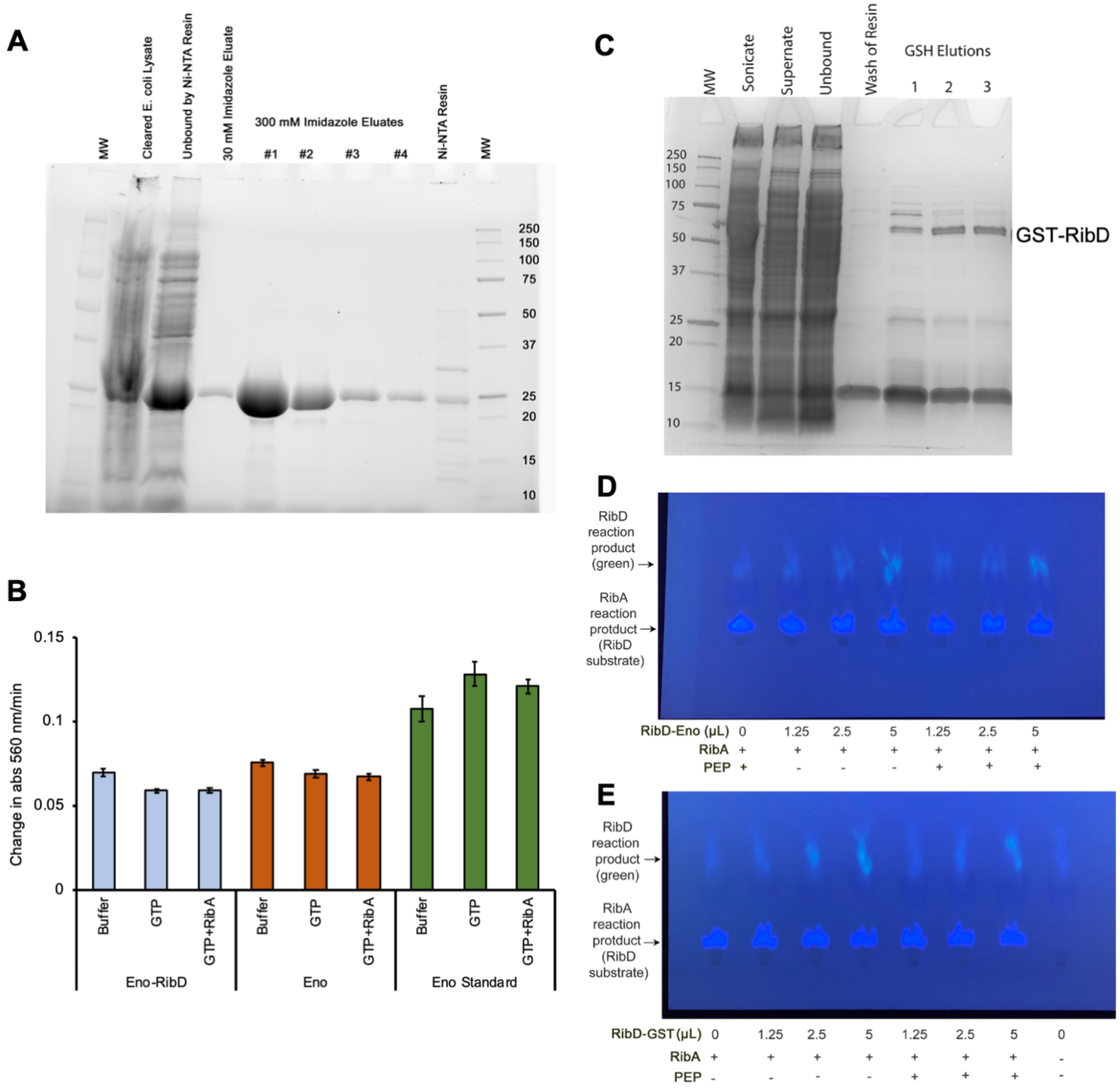

Figure S6. Enzyme activities of individual RibD and enolase proteins and their complex. **A.** Addition of RibD substrate (generated in situ by addition of GTP and purified *E. coli* RibA) has a minimal effect on enolase enzymatic activity whether or not it is in complex with RibD. Standard enolase (Sigma Millipore MAK178) is included as a positive control in both panels.) Data shown are means ± SEM of independent triplicates. Purified RibD-enolase (**B**) and GST-RibD (without enolase) (**C**) exhibit similar responses in enzymatic activity to addition of enolase substrate PEP. Quantification results are shown in Fig. 2G. **D**. Purification of *E. coli* histidine-tagged RibA by Nickel-affinity chromatography with evaluation of fractions by SDS-PAGE and stain-free UV imaging. **E.** Preparation of recombinant GST-tagged *F. novicida* RibD. *E. coli* expressing *F. novicida* GST-tagged RibD was sonicated, clarified by ultracentrifugation, applied to a glutathione-Sepharose column, and eluted with 10 mM GSH. Samples were applied to SDS-PAGE and visualized by staining with Coomassie Blue. The protein band corresponding to the predicted molecular weight of *F. novicida* GST-RibD is indicated.

Supplementary Movie

Movie S1. Morphing between the *Francisella* RibD dimer structure in the RibD-enolase complex and the *E. coli* RibD dimer structure (PDB ID 2OBC) showing that the two subunits are further apart in the *Francisella* structure.

REFERENCES

Abraham, M.J., Murtola, T., Schulz, R., Páll, S., Smith, J.C., Hess, B., and Lindahl, E. (2015). GROMACS: High performance molecular simulations through multi-level parallelism from laptops to supercomputers. SoftwareX 1, 19-25.

Afonine, P.V., Poon, B.K., Read, R.J., Sobolev, O.V., Terwilliger, T.C., Urzhumtsev, A., and Adams, P.D. (2018). Real-space refinement in PHENIX for cryo-EM and crystallography. Acta Crystallogr D Struct Biol 74, 531-544.

Bepler, T., Morin, A., Rapp, M., Brasch, J., Shapiro, L., Noble, A.J., and Berger, B. (2019). Positive-unlabeled convolutional neural networks for particle picking in cryo-electron micrographs. Nat Methods 16, 1153-1160.

Emsley, P., and Cowtan, K. (2004). Coot: model-building tools for molecular graphics. Acta Crystallogr D Biol Crystallogr 60, 2126-2132.

Graupner, M., Xu, H., and White, R.H. (2002). The pyrimidine nucleotide reductase step in riboflavin and F(420) biosynthesis in archaea proceeds by the eukaryotic route to riboflavin. J Bacteriol 184, 1952-1957.

Han, Y., Fan, X., Wang, H., Zhao, F., Tully, C.G., Kong, J., Yao, N., and Yan, N. (2020). High-yield monolayer graphene grids for near-atomic resolution cryoelectron microscopy. Proceedings of the National Academy of Sciences 117, 1009-1014.

Jo, S., Kim, T., Iyer, V.G., and Im, W. (2008). CHARMM-GUI: a web-based graphical user interface for CHARMM. Journal of computational chemistry 29, 1859-1865.

Kuhnel, K., and Luisi, B.F. (2001). Crystal structure of the Escherichia coli RNA degradosome component enolase. J Mol Biol 313, 583-592.

Mastronarde, D.N. (2005). Automated electron microscope tomography using robust prediction of specimen movements. J Struct Biol 152, 36-51.

Pettersen, E.F., Goddard, T.D., Huang, C.C., Meng, E.C., Couch, G.S., Croll, T.I., Morris, J.H., and Ferrin, T.E. (2021). UCSF ChimeraX: Structure visualization for researchers, educators, and developers. Protein Sci 30, 70-82.

Pfab, J., Phan, N.M., and Si, D. (2021). DeepTracer for fast de novo cryo-EM protein structure modeling and special studies on CoV-related complexes. Proc Natl Acad Sci U S A 118.

Punjani, A., Rubinstein, J.L., Fleet, D.J., and Brubaker, M.A. (2017). cryoSPARC: algorithms for rapid unsupervised cryo-EM structure determination. Nat Methods 14, 290-296.

Richter, G., Fischer, M., Krieger, C., Eberhardt, S., Lüttgen, H., Gerstenschläger, I., and Bacher, A. (1997). Biosynthesis of riboflavin: characterization of the bifunctional deaminase-reductase of Escherichia coli and Bacillus subtilis. J Bacteriol 179, 2022-2028.

Stenmark, P., Moche, M., Gurmu, D., and Nordlund, P. (2007). The crystal structure of the bifunctional deaminase/reductase RibD of the riboflavin biosynthetic pathway in Escherichia coli: implications for the reductive mechanism. J Mol Biol 373, 48-64.

Chai Discovery Team, Boitreaud, J., Dent, J., McPartlon, M., Meier, J., Reis, V., Rogozhonikov, A., and Wu, K. (2024). Chai-1: Decoding the molecular interactions of life. BioRxiv, 2024.2010. 2010.615955.

Wehrly, T.D., Chong, A., Virtaneva, K., Sturdevant, D.E., Child, R., Edwards, J.A., Brouwer, D., Nair, V., Fischer, E.R., Wicke, L., *et al.* (2009). Intracellular biology and virulence determinants of *Francisella tularensis* revealed by transcriptional profiling inside macrophages. Cell Microbiol 11, 1128-1150.
